# Communication skills in children aged 6–8 years, without cerebral palsy cooled for neonatal hypoxic-ischemic encephalopathy

**DOI:** 10.1038/s41598-022-21723-1

**Published:** 2022-10-22

**Authors:** Thomas J. Robb, James Tonks, Arthur P. C. Spencer, Sally Jary, Charlotte K. Whitfield, Marianne Thoresen, Frances M. Cowan, Ela Chakkarapani

**Affiliations:** 1 Bristol Medical School, Translational Health Sciences, University of Bristol, Bristol, UK; 2 Haven Clinical Psychology Practice, Cornwall, UK; 3 Division of Physiology, Institute of Basic Medical Sciences, University of Oslo, Oslo, Norway; 4 St Michael’s Hospital, Level D Neonatal Neuroscience, University Hospitals Bristol and Weston NHS Trust, Bristol, BS2 8EG UK

**Keywords:** Hypoxic-ischaemic encephalopathy, Neonatal brain damage, Language, Paediatric research, White matter injury

## Abstract

We assessed communication skills of 48 children without cerebral palsy (CP) treated with therapeutic hypothermia (TH) for neonatal hypoxic-ischemic encephalopathy (HIE) (cases) compared to 42 controls at early school-age and examined their association with white matter diffusion properties in both groups and 18-month Bayley-III developmental assessments in cases. Parents completed a Children’s Communication Checklist (CCC-2) yielding a General Communication Composite (GCC), structural and pragmatic language scores and autistic-type behavior score. GCC ≤ 54 and thresholds of structural and pragmatic language score differences defined language impairment. Using tract-based spatial statistics (TBSS), fractional anisotropy (FA) was compared between 31 cases and 35 controls. Compared to controls, cases had lower GCC (*p* = 0.02), structural (*p* = 0.03) and pragmatic language score (*p* = 0.04) and higher language impairments (*p* = 0.03). GCC correlated with FA in the mid-body of the corpus callosum, the cingulum and the superior longitudinal fasciculus (*p* < 0.05) in cases. Bayley-III Language Composite correlated with GCC (r = 0.34, *p* = 0.017), structural (r = 0.34, *p* = 0.02) and pragmatic (r = 0.32, *p* = 0.03) language scores and autistic-type behaviors (r = 0.36, *p* = 0.01).

## Introduction

Therapeutic hypothermia (TH) reduces mortality and neurodevelopmental disability in infants with neonatal hypoxic-ischemic encephalopathy (HIE) and is the standard treatment for HIE in the high-income countries^1,2^. However, children who undergo TH for HIE still have impaired cognition, especially verbal comprehension ability, with significantly lower verbal comprehension scores than age-matched controls even in the absence of cerebral palsy (CP)^3^. Given the association of verbal comprehension with communication^4^, children cooled for HIE might have language impairments.

Expressive and receptive language development in children cooled for HIE assessed using the Mullen Scales of Early Learning at 26 months of age^5^ indicated that expressive language scores were significantly lower than test norms, but receptive language scores were unaffected. Additionally, those children’s receptive language scores were associated with cortical/subcortical damage on neonatal brain MRI but there was no association between MRI lesions and expressive language scores. In another study, children without CP, who had been cooled for HIE, Bayley-III Language Composite scores at 30 months of age were found to be within the typical range, but the variability in the language scores correlated with peri-Sylvian brain volume on MRI at 6 months of age^6^. This study did not report expressive and receptive language scores. These studies suggest neonatal subcortical white matter injury with under-development at 6 months of age is associated with impaired language skills. Therefore, white matter microstructural development at early school-age may be associated with communication skills.

How communication evolves during childhood in children without CP who were cooled for HIE, how their communication skills differ from those of typically developing children, and whether there is an association between communication skills and brain microstructural development at early school-age are all unknowns. Therefore, we investigated the communication abilities of early school-age children without CP cooled for HIE (cases) and compared them to matched controls, examined associations between communication abilities with whole-brain white matter diffusion properties at early school-age and in cases, investigated the association between Language and Cognitive Composite scores at 18 months of age and communication abilities at early school-age.

## Methods

### Study design

This is a case–control study with ethics approval from the NRES Committee South West-Frenchay and Health Research Authority, UK (15/SW/0148) conducted in accordance with relevant guidelines and regulations between October 2015 and August 2019 at the University of Bristol. This was part of the CoolMRI study^4^, which is a case–control study examining the brain development and functional outcomes of children without CP who underwent TH for HIE, in comparison with control children matched for age, sex and socioeconomic status. No children in the study had a clinical diagnosis of autism spectrum disorder (ASD) as assessed by parental report in the CoolMRI study screening questionnaire. Two case children with hearing amplification devices for sensorineural hearing loss and one control child with error in completing the questionnaire were excluded. We obtained informed consent from parents and assent from children.

### Participants

The cases were children aged between 6 and 8 years without CP and were recruited from a population-based cohort of infants cooled for moderate to severe HIE between October 2007 and November 2012 according to regional TH guidelines^7^. All children were born at > 35 weeks gestation, were exposed to perinatal asphyxia, had evidence of moderate to severe encephalopathy assessed by neurological and amplitude integrated electroencephalogram assessment (aEEG) and did not have additional diagnoses. Controls, who were recruited via local schools in Bristol and newsletters circulated at the University of Bristol, included typically developing children who did not have neonatal problems and were matched at the group level with cases for age, sex and socio-economic status as determined by the index of multiple deprivation. We used a purposeful sampling approach to recruit case children and convenience sampling approach to recruit control children. All the case children, who underwent therapeutic hypothermia for neonatal HIE at St Michael’s hospital, Bristol, and were 6–8 years of age at the time of study, were invited to participate in the study. We announced the study at the schools in and around Bristol and at the University of Bristol to recruit control children. When families came forward to participate in the study, we screened for eligibility before recruiting them to the study. Index of multiple deprivation was calculated using the postcode where the child was living at early school-age and was based on the weighted combination of seven domains of deprivation including income, employment, education housing, health, disability and crime (1 most deprived; 10 least deprived) as defined by the UK Government, which is applicable to England^8^.

Baseline demographic and clinical information was collected including sex, birth weight, gestational age at birth, and weight, height and head circumference at school age examination. Parents provided information on the need for a statement of special educational needs, additional support at school or educational psychology support at any time during childhood. For cases, we collected additionally Apgar score at 10 min, worst pH and base excess within 1 h after birth, need for ventilation at 10 min, clinical severity of encephalopathy and pattern of aEEG before commencing TH.

#### Cases

All case children had regular follow-up until 24 months of age including the assessment of motor, cognition and language skills using the Bayley Scales of Infant and Toddler Development version III (Bayley-III) at 18 months of age. Bayley-III generates distinct Cognitive Composite, Language Composite, and Motor Composite scores from raw scores, which have a normative mean (SD) of 100 (15). A Bayley-III Language or Cognitive Composite score < 85 was used to denote impairment^9^. Cerebral palsy was ruled out at 2 and 6–8 years of age after a standard neurological examination including assessment of motor function, muscle tone and deep tendon reflexes performed by either a consultant paediatrician, or experienced paediatric physiotherapist.

#### Cases and controls

The parents/caregivers completed the Children’s Communication Checklist Second Edition (CCC-2)^10^ on the same day that the children underwent MRI without sedation at the Clinical Research and Imaging Centre, University of Bristol, UK (CRiCBristol). The CCC-2 is a parental/carer report questionnaire for discriminating between typically developing children and those with clinically significant communication difficulties aged 4 to 16.9 years. It has been validated against the psychologist-administered Wechsler Abbreviated Scales of Intelligence (WISC), the British Picture Vocabulary Scales and the Clinical Evaluation of Language Fundamentals-III (CELF-III)^10^. All children fulfilled the CCC-2 inclusion criteria of using English as their primary language, speaking in full sentences and having no permanent hearing loss.

The CCC-2 consists of 70-items that generate 10 standardised (age-scaled) subscales that assess three different aspects of language function. Subscales including Speech, Syntax, Semantics and Coherence measure aspects of structural language. pragmatic language is assessed by the subscales: Inappropriate Initiation, Stereotyped Language, Context and Non-Verbal Communication. The subscales, Social Relations and Interests represent current autistic type behaviours. Each subscale consists of seven items that measure how often a behaviour is observed on a four-point scale (0 = less than once a week, 1 = at least once a week, but not every day, 2 = once or twice a day, 3 = more than twice a day, 4 = always). Where ≥ 2 questions within a given subscale are not answered, this subscale is excluded from analysis. For each subscale, raw scores were converted to a normative age-standardised score (mean 10, SD 3), whereby higher scores indicate better performance. Summation of scores for the 8 subscales of structural and pragmatic language results in a General Communication Composite (GCC) score. The mean (SD) GCC of a UK standardisation cohort is 80 (15) and a GCC score of ≤ 54 indicates the 10th percentile and likely clinically significant language difficulties^10^. A ‘Social Interaction Difference Composite’ (SIDC) score was calculated by subtracting the sum of structural language subscales from the sum of pragmatic language subscales to identify children with disproportionate structural/ pragmatic language deficits. Four different language/communication profiles are defined as: typical language (GCC ≥ 55 and SIDC > -15); structural language impairment (GCC ≤ 54 and SIDC ≥ 9); intermediate group (GCC ≤ 54 and SIDC between 0 and 8) and pragmatic language impairment (GCC ≤ 54 and SIDC < 0, or SIDC ≤ -15 irrespective of GCC)^11^. The intermediate group suggests the possibility of either structural or pragmatic language impairment or both. Children with language impairment was defined to have either structural, intermediate or pragmatic language impairment.

### MRI acquisition and preprocessing

Diffusion-weighted imaging (DWI) data were acquired, as previously reported^12^, with a Siemens 3 T Magnetom Skyra MRI scanner at CRiCBristol. An experienced radiographer placed children supine within the 32-channel receive only head-coil, and head movement was minimised with memory-foam padding. Children wore earplugs and were able to watch a film of their choice. DWI data were acquired with a multiband echo-planar imaging (EPI) sequence, using the following parameters: TE = 70 ms; TR = 3150 ms; FoV 192 × 192 mm; 60 slices; 2.0 mm isotropic voxels, flip angle 90°, phase encoding in the anterior–posterior direction, in-plane acceleration factor = 2 (GRAPPA)^13^, through-plane multi-band factor = 2^14,15^. For the purpose of data averaging and eddy-current distortion correction, two sets of diffusion-weighted images were acquired with b = 1000 s mm^−2^ in 60 diffusion directions, equally distributed according to an electrostatic repulsion model, as well as 8 interspersed b = 0 images, with one data set acquired with positive phase encoding steps, then repeated with negative steps (so-called, “blip-up, blip-down”), giving a total of 136 images. DWI data were corrected for eddy current induced distortions and subject movements using EDDY^16^ and TOPUP^17^ from the FMRIB Software Library (FSL, http://fsl.fmrib.ox.ac.uk)^18^. The quality of the DWI data was assessed using the EddyQC tool^19^ from FSL, which gives metrics indicating the level of movement and eddy currents in each direction. Scans were rejected if the root-mean-square of these metrics was greater than one standard deviation above the mean for the whole cohort.

### White matter analysis

Fractional anisotropy (FA) is a measure of the directionality of diffusion of water molecules through the brain and is affected by properties of brain tissue such as myelination and fibre density^20^. To examine the association between white matter microstructure and communication abilities, we used tract-based spatial statistics (TBSS)^18^ to measure the correlation between white matter FA and GCC score in cases and controls separately, as follows.

Subject-specific FA images were generated by fitting a tensor model to the DWI data using the weighted least squares method in FSL’s FDT software. Within each group, all subjects were nonlinearly registered to one subject (following the recommended procedure for testing data from children), chosen automatically by finding the most representative subject, which was then affine registered to MNI standard space. A threshold of 0.3 was then used to create a skeletonised representation of the white matter tracts. Each subject’s registered FA image was then projected onto this skeleton to allow voxelwise statistics.

### Statistical analysis

Primary outcome was GCC and secondary outcomes were structural language score, pragmatic language score, current autistic-type behaviors, language profiles, association between white matter FA and GCC, Bayley-III Language and Cognitive Composite score at 18 months and GCC, structural, pragmatic language or autistic-type behavior scores. Normality of data was established using Shapiro–Wilk’s test. Homogeneity of variance was explored using Levene’s test. Two-tail between group differences (cases vs controls) in GCC score was analysed using a students unpaired T-test and graphically represented as bars (mean, SD). Two-tail between groups differences (cases vs controls) in structural language, pragmatic language and autistic-type behaviours scores were analysed using Mann–Whitney-U tests and graphically represented as violin plots (median, IQR).

Categorical variables are presented as proportions. Between-groups differences in categorical data were analysed using N-1 Chi-Square test. Associations between GCC, structural and pragmatic language scores and autistic-type behavior scores at 6–8 years, and Bayley-III Language and Cognitive Composite scores, and GCC and deprivation index were examined using Pearson correlations, as data were normally distributed. Given that structural and pragmatic language scores are components of GCC, correction for multiple comparisons was not applied for case–control differences and associations with Bayley-III scores in cases.

For TBSS, we assessed the correlation between voxelwise FA and GCC score, in cases and controls separately, with age and sex included as covariates in a general linear model. Significance was tested using FSL’s non-parametric permutation testing software, RANDOMISE^21^. We used 10,000 permutations and applied threshold-free cluster enhancement (TFCE) to correct for multiple comparisons. Significant results have corrected p < 0.05.

Statistical analyses were performed using IBM SPSS Statistics for Windows, Version 25, Armonk, NY: IBM Corporation and GraphPad Prism version 8.0.0 for Windows (GraphPad Software, San Diego, California USA, www.graphpad.com). Two-tailed *p* < 0.05 was considered statistically significant.

## Results

**Figure 1:**
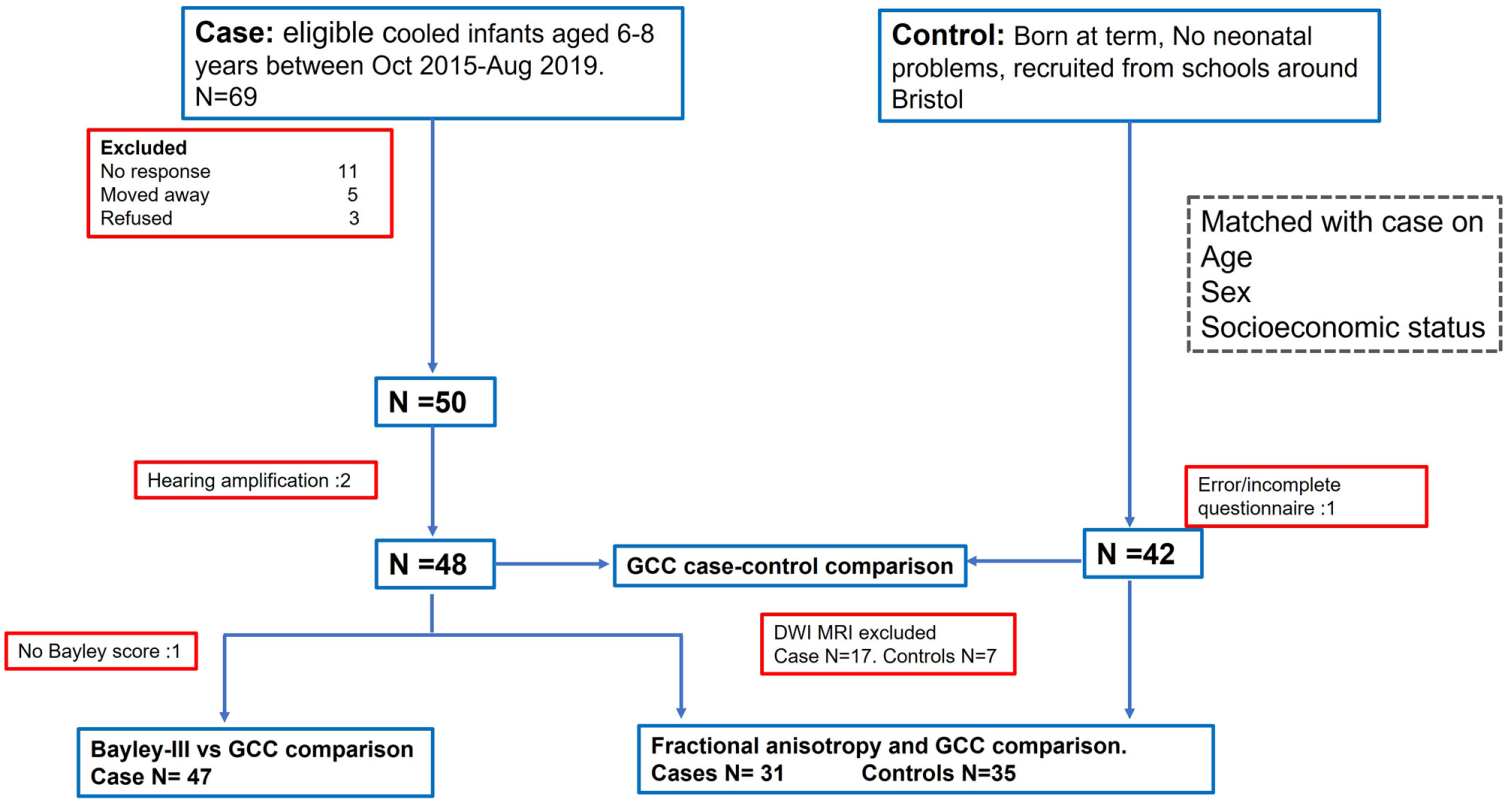
Study recruitment flowchart indicating exclusion criteria at each stage of recruitment, and subsequent N number of participants included in analyses of: GCC case–control comparison, Fractional anisotropy and GCC correlation in cases and in controls, and Bayley-III–GCC correlation in cases. For MRI analysis, participants were excluded who did not undergo MRI, or where significant movement artefact was present.

The study recruitment flow chart is shown in Fig. 1. Of the 48 cases and 42 controls included in the CCC-2 analysis, 31 cases and 35 controls had useable DWI data. Anatomical images for participants included in the MRI analyses were visually assessed by an assessor (FC) blinded to case/control status for focal lesions and abnormal signal intensities. Lesions were present in 1 case and 2 controls, and were judged to be non-severe and therefore were not excluded. There were no significant differences in demographics between cases and controls included in the main analysis or in the MRI-communication score association analysis (Table 1). Patient characteristics were comparable between those included in MRI analysis and those who were rejected from MRI analysis (Supplementary Table S3). Factors associated with those rejected from MRI analysis in this cohort are explored in detail in Woodward et al.^22^.

**Table 1:** Baseline demographics for cases and controls.

Demographics	CCC-2 Questionnaire			White Matter Analysis		
	Cases (N = 48)	Controls (N = 42)	P value	Case (N = 31)	Controls (N = 35)	P value
Assessment age in months, mean (SD)	84.1 (5.9)	84.7 (6.3)	0.63	83.1 (5.9)	84.2 (6.7)	0.47
Female sex, N (%)	19 (39.6%)	20 (47.6%)	0.52	15 (48.4%)	16 (45.7%)	1.0
Index of multiple deprivation decile, median (IQR)	7 (4–8.75)	7 (5–9)	0.15	7 (4–9)	7 (5–8.75)	0.45
Gestational age in weeks, median (IQR)	40.1 (38.8–40.9)	40.2 (39.5–41.2)	0.16	40.4 (39.0–41.0)	40.3 (39.5–41.3)	0.19
Birth weight in grams, median (IQR)	3352 (3092–3780)	3552 (3111–3928)	0.35	3362 (3113–3895)	3510 (3090–3886)	0.81
Weight at early school-age in kg, median (IQR)	24.9 (22.0–27.8)	23.4 (22.2–28.0)	0.46	25.1 (21.8–28.3)	23.2 (22.1–27.9)	0.52
Height at early school-age in cm, median (IQR)	125 (120–130)	124 (120–130)	0.59	123 (118–129)	124 (120–130	0.68
Head circumference at early school-age cm, median (IQR)	52.5 (51.55–53.45)	52.5 (51.5–53.0)	0.60	52.5 (51.03–53.08)	52.5 (51.52–53.00)	0.89

### Primary outcome

**Figure 2:**
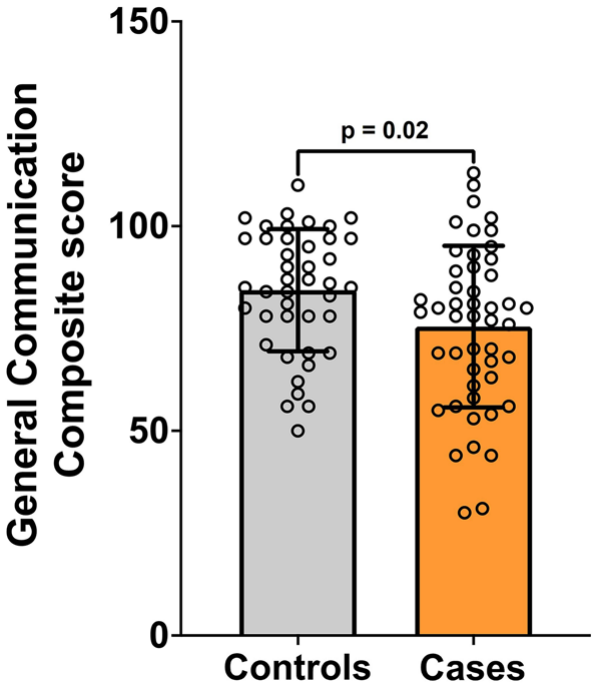
Comparison of mean General Communication Composite score in control (gray) and case (orange) children.

Case children had significantly lower mean (SD) GCC score, and a wider range of scores, than controls [75.5 (19.75) vs 84.4 (14.94), *p* = 0.02] (Fig. 2). Whilst there was no impact of age or sex on GCC in cases and controls, the deprivation index was positively correlated with GCC in cases (r = 0.31, *p* = 0.03) but not in controls (r = 0.01, *p* = 0.9) (Supplementary Fig. S1).

### Secondary outcomes

**Figure 3:**
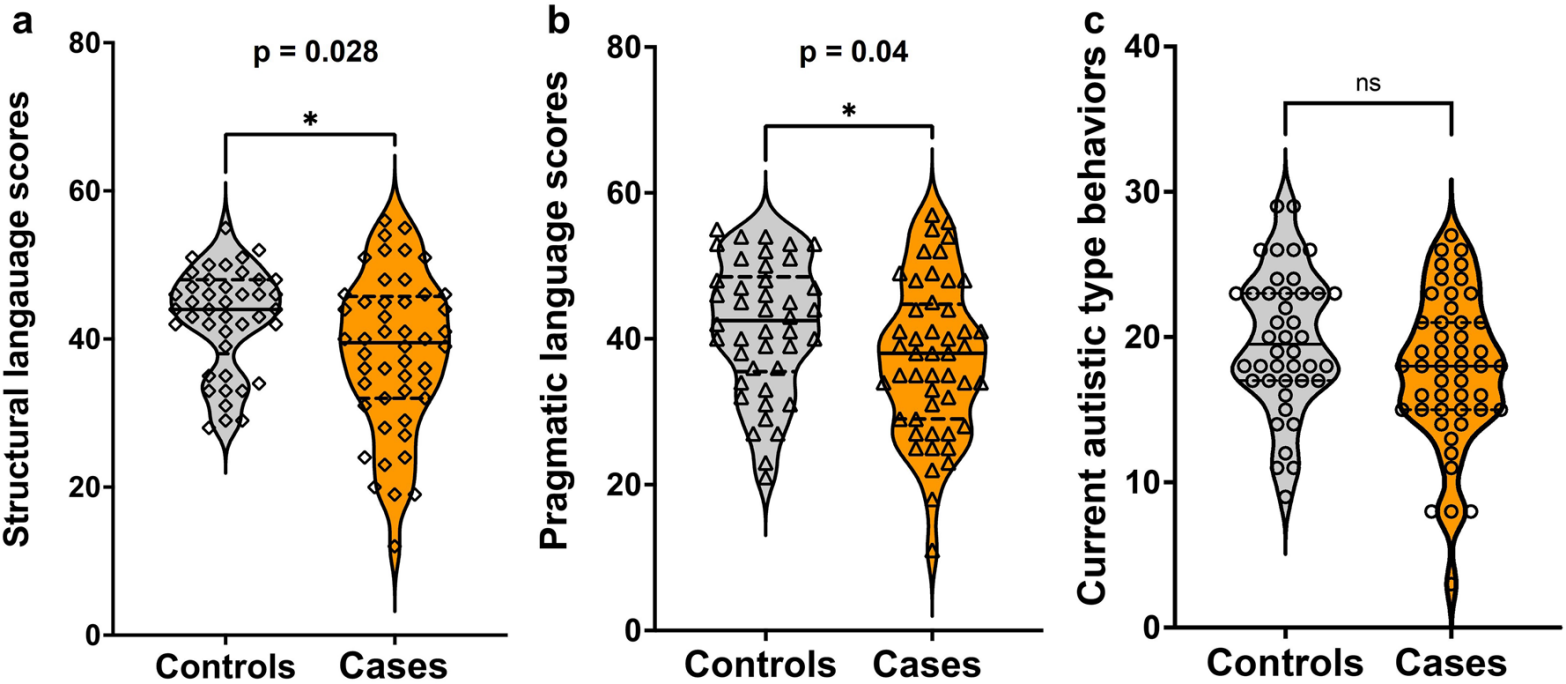
Comparison of structural language scores (**a**), pragmatic language scores (**b**) and autistic type behaviors (**c**) between control (gray) and case (orange) children.

Median (IQR) structural language score was significantly lower in cases than controls [39.5 (32.0, 45.75) vs 44.0 (38.0, 48.0), *p* = 0.028] (Fig. 3a). Cases had significantly lower median (IQR) pragmatic language score than controls [38 (29, 44.75) vs 42 (35.5, 48.5), *p* = 0.04] (Fig. 3b). The difference in the current autistic type behaviors between cases and controls was not significant (Fig. 3c). The subscale scores of structural and pragmatic language and current autistic behaviors are given in Supplementary Table S1. The pragmatic and structural language scores were significantly positively correlated in cases (r = 0.77, p < 0.0001) and controls (r = 0.74 p < 0.0001) (Supplementary Fig. S2). The SIDC did not differ between cases and controls but the distribution of SIDC indicates that the structural language scores were lower than the pragmatic language scores in cases and controls. (Supplementary Fig. S3).

When the communication scores were classified into clinically defined language impairments, 16.7% (8/48) cases had language impairments compared to 2.3% (1/42) controls, *p* = 0.03, (Table 2). Children with language impairments had significantly higher need for help at school than children without language impairments (44.4% (4/9) vs 14.8% (12/81), *p* = 0.03).

**Table 2:** Language and communication profiles of case and control children.

Language/communication profile	CCC-2 questionnaire		White matter analysis	
	Cases (N = 48)	Controls (N = 42)	Cases (N = 31)	Controls (N = 35)
Typical language, N (%)	40 (83.3%)	41 (95.3%)	27 (87.1%)	34 (97.1%)
Any language impairment N (%)	8 (16.7%)	1 (2.3%)	4 (12.9%)	1 (2.9%)
Structural language impairment, N (%)	1 (2.0%)	0 (0%)	0 (0%)	0 (0%)
Intermediate group, N (%)	3 (6.1%)	0 (0%)	3 (9.7%)	0 (0%)
Pragmatic language impairment, N (%)	4 (8.2%)	1 (2.3%)	1 (3.2%)	1 (2.9%)

### White matter analysis

**Figure 4:**
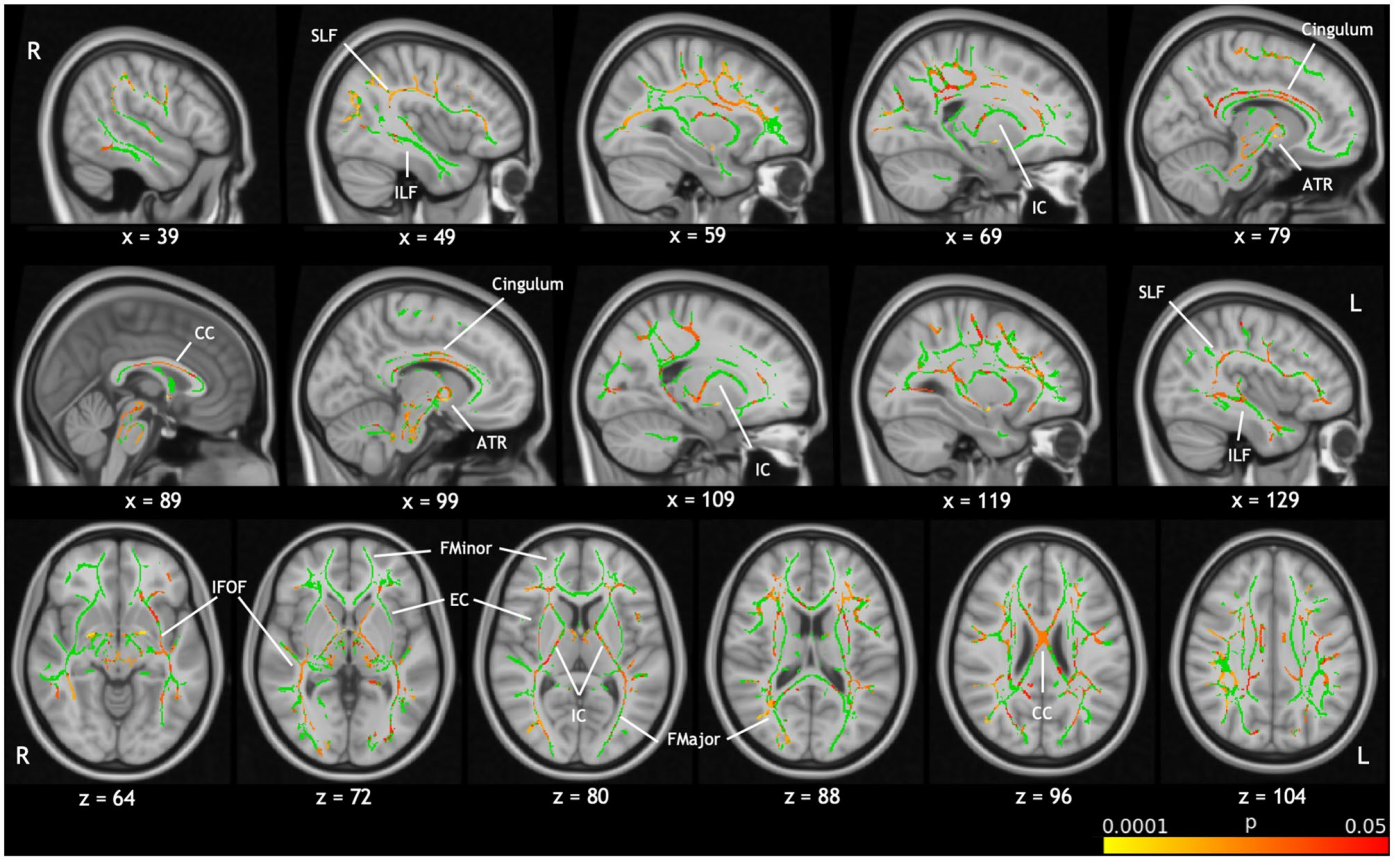
TBSS results showing areas of white matter which exhibit correlation between FA and GCC score in cases, independent of age sex and deprivation index (p < 0.05, TFCE-corrected). No significant correlations were found in controls. The white matter skeleton is shown in green with significance of positive correlations indicated by the colour bar. These are overlaid on the MNI standard template with the position of the slice in MNI space given under each slice. Labels indicate some major white matter tracts and regions. *ATR* anterior thalamic radiation, *CC* corpus callosum, *EC* external capsule, *FMajor* forceps major, *FMinor* forceps minor, *IC* internal capsule, *IFOF* inferior fronto-occipital fasciculus, *ILF* inferior longitudinal fasciculus, *SLF* superior longitudinal fasciculus.

Figure 4 shows areas of white matter in which FA correlated with GCC in the case group, independent of age and sex, as determined by TBSS (TFCE-corrected p < 0.05). Correlations were found in widespread areas of white matter, including the mid-body of the corpus callosum, the cingulum and the superior longitudinal fasciculus (SLF). There were no significant correlations in the control group.

### Relationships between perinatal characteristics, Bayley-III Language and Cognitive Composite score and GCC in cases

In case children, there were no significant differences in the severity of asphyxia defined by pH or base excess in the first hour after birth, Apgar score at 10 min, and clinically assessed severity of encephalopathy or aEEG abnormalities before commencing TH between children with and without language impairments (Supplementary Table S2).

**Figure 5:**
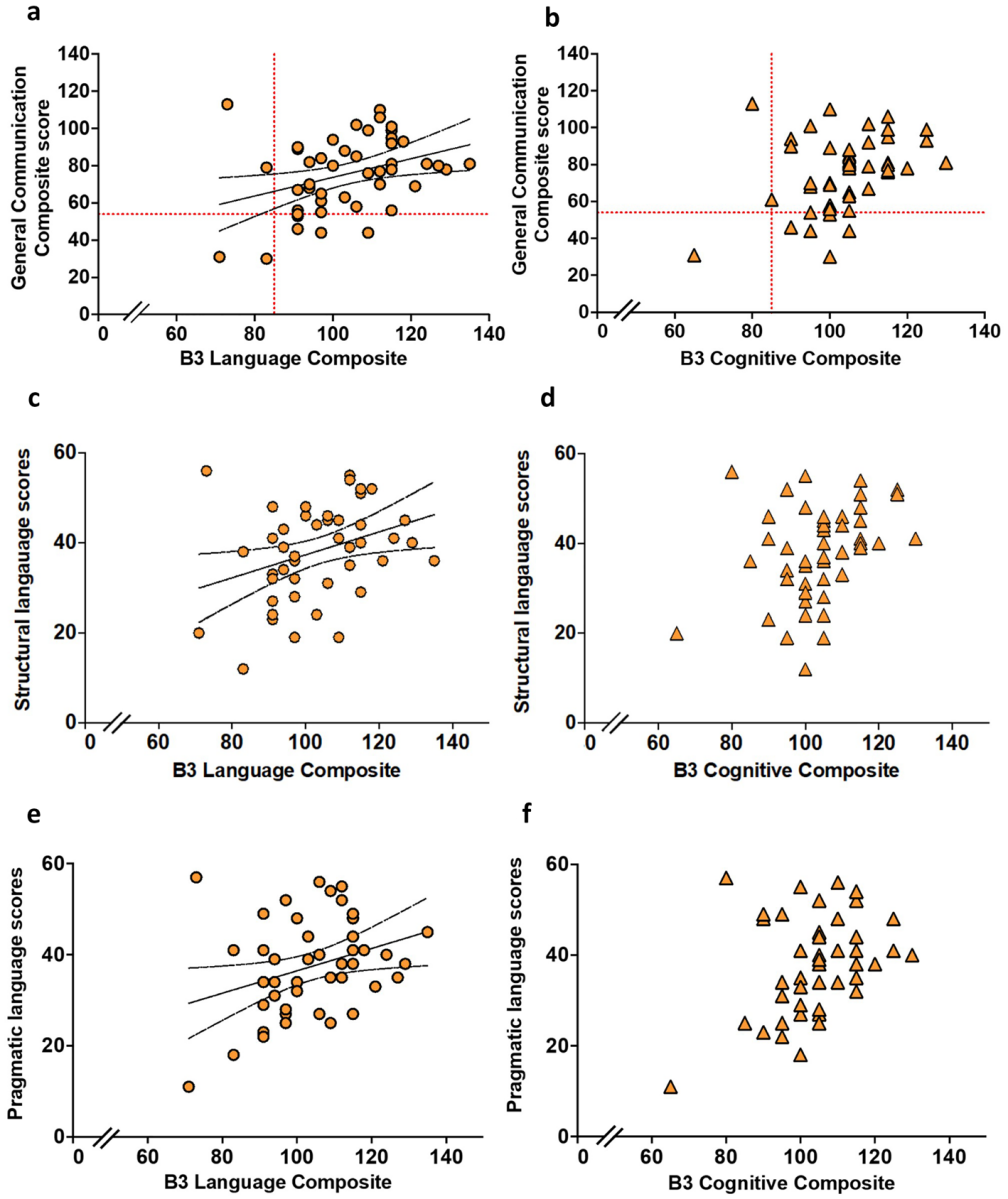
Comparison of Bayley-III Language Composite scores and GCC (**a**); structural language (**c**); pragmatic language (**e**). Comparison of Bayley-III Cognitive Composite scores and GCC (**b**); structural language (**d**); pragmatic language (**f**). The Y-axis dashed line in figure (**a**) and (**b**) represents GCC = 54 (10th centile of the UK standardised cohort). The X-axis dashed line in figure (**a**) and (**b**) represents Bayley-III Composite score = 85 (1 SD below the normative mean).

**Figure 6:**
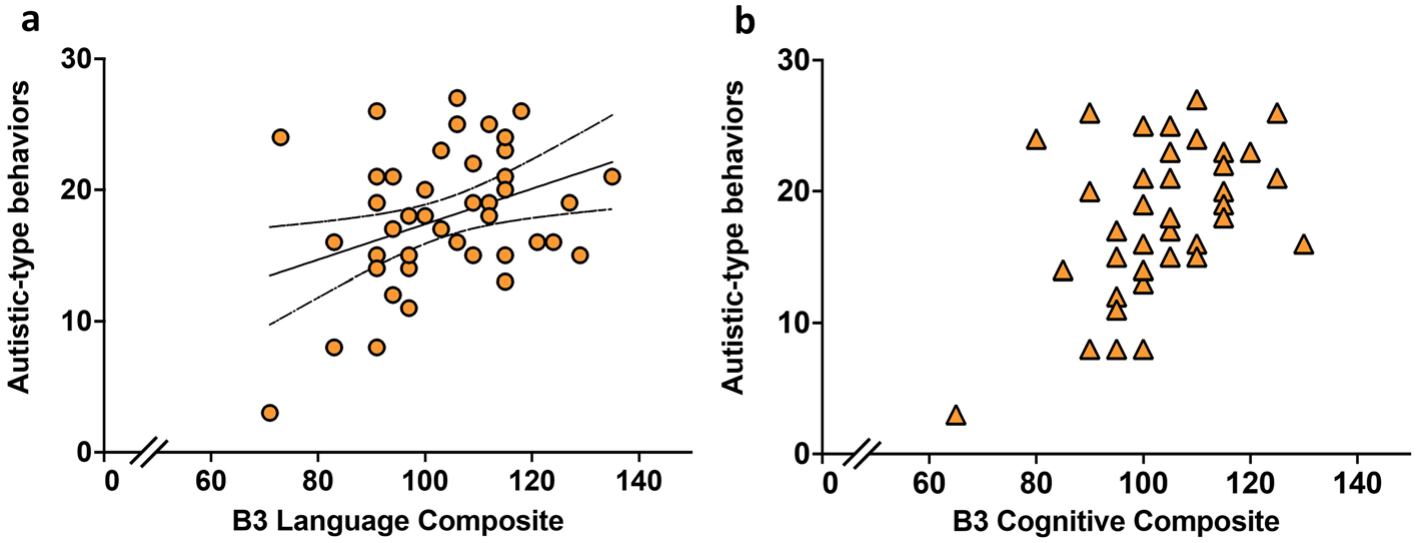
Comparison of autistic-type behaviors scores and Bayley-III Language (**a**) and Cognitive (**b**) Composite scores.

Of 48 case children, one child did not have Bayley-III Cognitive and Language Composite scores. There were positive linear associations between Bayley-III Language Composite and CCC-2 GCC scores (r = 0.35, *p* = 0.017) (Fig. 5a), and its components, structural (r = 0.34, *p* = 0.02) (Fig. 5c) and pragmatic language scores (r = 0.32, *p* = 0.03) (Fig. 5e). Autistic-type behavior scores were positively correlated with Bayley-III Language scores (r = 0.36, *p* = 0.01) (Fig. 6a). There was no linear relationship between Bayley-III Cognitive Composite score and GCC, its components, structural and pragmatic language scores or autistic-type behavior scores (Figs. 5b,d,f, 6b). Bayley-III Language Composite score < 85 predicted GCC ≤ 54 with a positive predictive value of 50% and negative predictive value of 88.4%. 5/43 (11.6%) children with Bayley-III Language Composite score > 85 at 18 months of age had an impaired GCC score of ≤ 54 at 6–8 years of age, and 2/4 (50%) of children who had Bayley-III Language Composite score < 85 at 18 months of age had a non-impaired GCC of > 55 at 6–8 years of age (Fig. 5a).

## Discussion

Early-school aged children without CP who were cooled for HIE have significantly lower GCC scores including its components, structural and pragmatic language scores compared to matched controls assessed using parental report. Using the CCC-2 as a measure of language ability, case children compared with controls had greater language impairments accompanied by need for support at school. In cases, 18-month Bayley-III Language Composite scores were associated with GCC, structural and pragmatic language scores and autistic-type behaviors. Further, FA in widespread white matter was associated with GCC independent of age and sex, including the mid-body of the corpus callosum, the cingulum and the SLF. No correlations were found in controls.

The UK standardised cohort has a mean GCC of 80. Mean GCC in our control group was 84.4, whilst in the case group was 75.5; significantly below the control group and the population mean^10^. The reduction in GCC in cases compared with controls was contributed to by lower scores across both components of GCC, structural and pragmatic language scores. This resulted in 17% of case children characterised as having language impairments including either structural or pragmatic language impairment, or being in an intermediate group. Children previously cooled for HIE have impairments in pragmatic language (expressive language and non-verbal communication) at 2 years of age, whilst exhibiting preserved receptive language scores^5^. Another study of children without CP previously cooled for HIE found evidence of structural language impairment at 5 years of age as measured by the CELF-2^23^. In non-cooled survivors of HIE with basal ganglia and thalamic injury, communication impairments reported by parents were present in 82% of children with CP, and in 30% of children without CP at 2 years^24^. Children without CP, previously cooled for HIE had verbal IQ scores below the normative mean (100) at 4 years of age [85.9 (19.1)]^3^. We have previously shown in this cohort that cases had lower verbal IQ scores than matched controls at 6–8 years of age [94 (8.79) vs 103 (10.09)], as measured by the Wechsler Intelligence Scales for Children, Fourth UK Edition (WISC-IV)^4^. Our current results therefore support the view that despite TH, the language ability in these children is adversely affected at early school age.

Pragmatic communication continues to develop through the early school years corresponding to the structural and functional development of medial prefrontal and lateral temporo-parietal brain regions that are involved in pragmatic communication^25–27^. Further, a diffuse insult to frontal and temporal lobes and the corpus callosum at an earlier age might impact the development of pragmatic language skills^28^. Brain imaging studies in neonates cooled for HIE have reported injury to corpus callosum^29^ which might impact the development of networks involving the temporal lobes that are recruited during pragmatic language functions, affecting the development of pragmatic language skills in these children^30^. Additionally, the cingulate cortices (with right-sided predominance) are thought to play a key role in pragmatic language^31,32^. Studies have also demonstrated the importance of the SLF in higher-order language function^33,34^. We found correlation between FA and GCC score, in the case group, in widespread areas of white matter including the corpus callosum, cingulum and SLF. Our results therefore support a neuroanatomical correlate for language impairment in these children. While diffusion properties in several white matter tracts have been linked to language abilities in healthy controls^35,36^, we found no correlations between GCC and FA in the control group, possibly due to the small sample size. The presence of significant correlations in the similarly small case group demonstrates a strong association between white matter diffusivity and language abilities.

We have previously reported, in the same cohort, reduced FA in many areas of white matter in cases compared to controls, including the corpus callosum and cingulum^12^. This reduction in FA reflects underlying microstructural alterations which may impose limitations on the cognitive abilities of these children, resulting in the association between communication scores and FA in these regions.

Autistic-type behavior scores did not significantly differ between cases and controls. However, the mean interests subscale score included within the autistic-type behaviours was significantly lower in cases than controls. However, the case children mean interests score (8.7) was not as low as that found in children with autism (3.36)^10^. Therefore, the case children in this study may not reach the threshold for the diagnosis of autistic spectrum disorder. Future studies in children cooled for HIE should include detailed multidimensional assessment for diagnosing Autism Spectrum Disorder.

In cases, language abilities assessed using Bayley-III scales at 18 months correlated with the communication and autistic-type behavior scores at early school-age as reported by parents. The lateralisation of cortical language centres develop by 5 years of age^37^ and is refined throughout adolescence^38^. Different modes of perinatal brain injury may differentially affect maturation of these regions. Neuroplastic recovery of many cognitive functions, including expressive language, has been shown to occur in perinatal stroke by 7–11 years of age^39–42^. Neuroplastic recovery of language skills may not be as complete in more diffuse, global perinatal brain injuries such as in HIE, or fetal growth restriction. Using the CCC-2, children with fetal growth restriction were reported to have lower GCC scores compared to matched controls. Sub-analysis revealed that these differences were only present in children > 9 years of age, suggesting that, in contrast to children with a history of perinatal stroke, language impairments in children with fetal growth restriction become more pronounced with age^43^. In the present study, of children with Bayley-III Language scores within the normative range at 18 months (scores > 85), nearly 12% had GCC scores < 55 (10th percentile) at 6–8 years, which may suggest that communication impairments become more evident as children grow and also highlights the limitation of early language assessment in predicting future communication impairments.

Socioeconomic deprivation has been shown to independently affect language and communication development in children^44^. We found that socioeconomic status as measured by the index of multiple deprivation was associated with GCC scores in cases, but not in controls. It is possible that the communication skills of case children are differentially affected by deprivation compared to controls. This has also been observed in preterm cohort with infants from most deprived neighbourhoods having a three-fold increased odds of speech, language and communication problems compared with preterm infants from least deprived neighbourhoods^45^.

### Limitations

Including case children with CP may have allowed us to capture a wider profile of language impairments associated with HIE. However, nearly three-quarters of survivors who were cooled for HIE in contemporary cohorts do not have CP^46^. Therefore, our findings are more pertinent to the current real-world practice. Movement during MRI scanning is a common difficulty when scanning children of this age group. We used a thorough automated quality control pipeline to reject scans with excessive movement. However, this further limited the sample size for the MRI analysis.

In conclusion, we found that early school-aged children without CP previously cooled for HIE demonstrate lower communication scores than matched controls, indicating often unrecognised communication difficulties. We identified brain structural correlates of communication ability in case children, which were not present in controls. Additionally, communication abilities in case children were associated with the UK index of deprivation, and language scores obtained from clinical assessments at 18 months of age. Communication difficulties in children cooled for HIE may be a barrier to ongoing social and educational development in both the short and long-term.

## Supplementary Information

Supplementary Information 1.

Supplementary Information 2.

Supplementary Information 3.

Supplementary Information 4.

## Data Availability

The data that support the findings of this study are available upon request from the corresponding author (EC).
