# Supplementary figures and images for: Communication skills in children aged 6–8 years, without cerebral palsy cooled for neonatal hypoxic-ischemic encephalopathy
Source: Sci Rep. 2022 Oct 22;12:17757. doi: 10.1038/s41598-022-21723-1 (PMC9588000; doi:10.1038/s41598-022-21723-1)

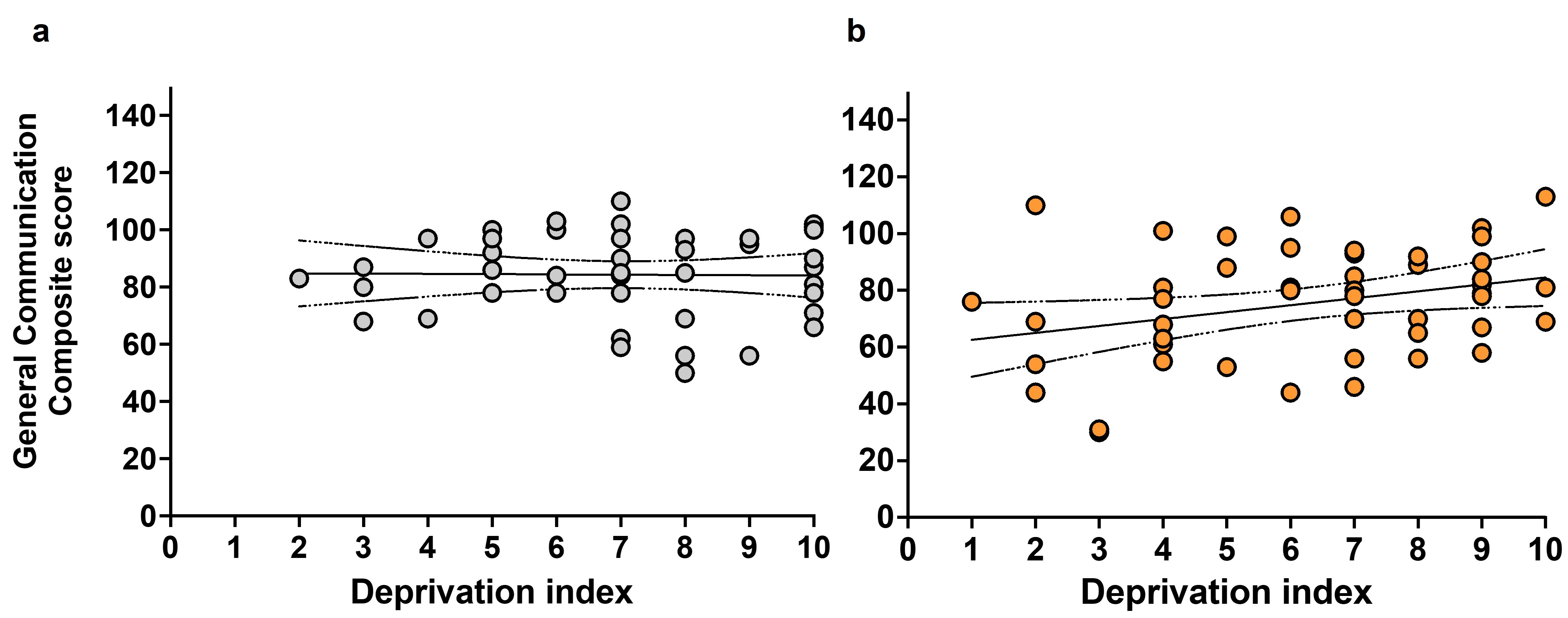

Supplement: Supplementary file 1 — Supplementary Information 1. [file 41598_2022_21723_MOESM1_ESM.jpg]

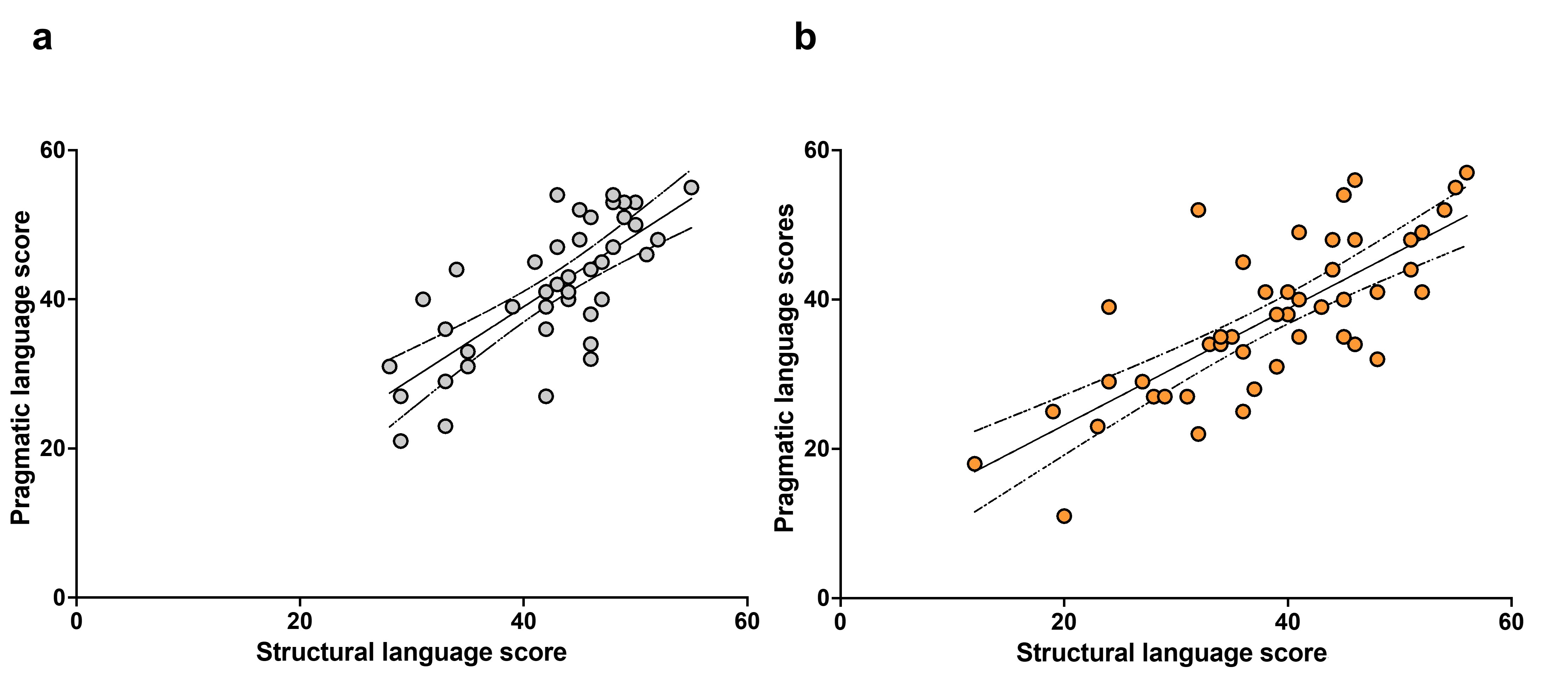

Supplement: Supplementary file 2 — Supplementary Information 2. [file 41598_2022_21723_MOESM2_ESM.jpg]

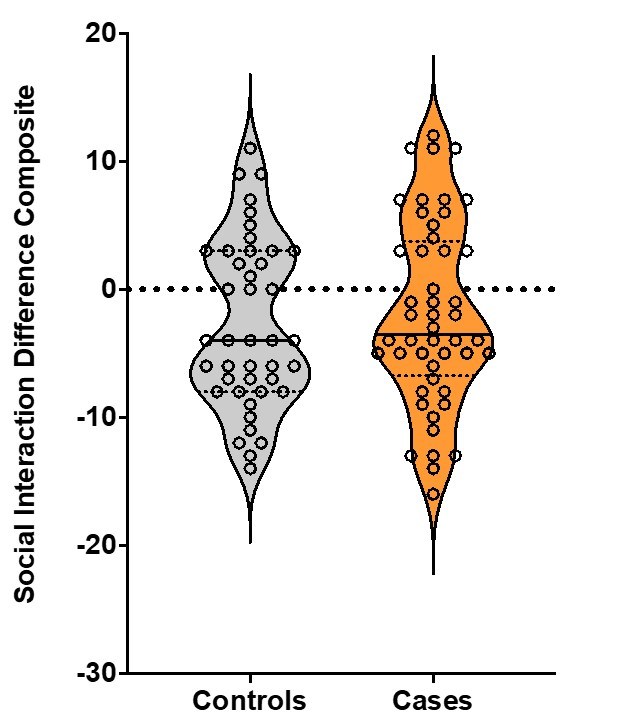

Supplement: Supplementary file 3 — Supplementary Information 3. [file 41598_2022_21723_MOESM3_ESM.jpg]
