# Supplementary material for: Communication skills in children aged 6–8 years, without cerebral palsy cooled for neonatal hypoxic-ischemic encephalopathy
Source: Sci Rep. 2022 Oct 22;12:17757. doi: 10.1038/s41598-022-21723-1 (PMC9588000; doi:10.1038/s41598-022-21723-1)
Supplement: Supplementary file 4 — Supplementary Information 4. [file 41598_2022_21723_MOESM4_ESM.docx]

Supplementary Table 1: Subscales of Children’s Communication Checcklist-2 in cases and controls

|  |  | **Mean (SD) score** | | | |
| --- | --- | --- | --- | --- | --- |
|  |  | **CCC-2 Questionnaire** | | **White Matter Analysis** | |
| **Language Domain** | **CCC-2 subscales** | **Cases (N=48)** | **Controls**  **(N =42)** | **Cases (N=31)** | **Controls (N=35)** |
| Structural Language | Speech | 9.8 (3.34) | 11.1 (2.22) | 9.8 (3.45) | 11.1 (2.17) |
|  | Syntax | 9.7 (3.10) | 11.2 (2.13) | 9.5 (3.45) | 11.3 (2.05) |
|  | Semantic | 9.2 (3.44) | 10.6 (2.68) | 9.1 (3.50) | 10.8 (2.72) |
|  | Coherence | 9.4 (3.43) | 9.9 (2.69) | 9.0 (3.24) | 9.9 (2.82) |
| Pragmatic Language | Inappropriate Initiation | 9.4 (3.12) | 10.7 (2.75) | 9.5 (3.36) | 10.7 (2.76) |
|  | Stereotyped | 9.8 (3.17) | 10.8 (2.70) | 9.6 (3.17) | 11.1 (2.64) |
|  | Context | 9.1 (3.39) | 10.5 (3.14) | 9.1 (3.27) | 10.8 (3.04) |
|  | Nonverbal | 9.0 (3.13) | 9.6 (3.03) | 9.1 (2.83) | 9.9 (3.02) |
| Autistic-type behaviours | Social | 9.1 (3.55) | 9.7 (2.99) | 9.0 (3.35) | 9.9 (3.06) |
|  | Interests | 8.7 (2.40) | 10.0 (2.63) | 8.6 (2.24) | 10.3 (2.70) |

Supplementary Table 2: Perinatal characteristics of asphyxia and encephalopathy in case children with and without language impairment

| Variable | Cases with language impairment (N = 8) | Cases with GCC >55  (N = 40) |
| --- | --- | --- |
| Worst pH within 1 hour of birth, median (IQR) | 6.9 (6.80-7.01) | 6.96 (6.81-7.12) |
| Worst BE within 1 hour of birth, median (IQR) | -16.9 (-23.2, -11.8) | -16.1 (-22.4, -12.0) |
| Apgar score at 10 min, median (IQR) | 6 (5-8) | 7 (5-8) |
| Need for ventilation at 10 minutes, N (%) | 6 (75%) | 27 (67.5%) |
| aEEG abnormalities before TH  Moderately abnormal, N (%)  Severely abnormal, N (%) | 8 (100%)  0 (0%) | 38 (95%)  2 (5%) |

Supplementary Table 3: Comparison of clinical characteristics at birth between cases included or rejected from MRI analysis.

| **Demographics** | **Included Cases**  **(N=31)** | **Rejected Cases**  **(N=17)** | **P value** |
| --- | --- | --- | --- |
| Gestational age in weeks, median (IQR) | 40.4 (39.0-41.0) | 39.7 (38.3-40.8) | 0.34 |
| Birth weight in grams, median (IQR) | 3362 (3113-3895) | 3250 (3025-3690) | 0.38 |
| Worst pH within 1 hour of birth, median (range) | 6.98 (6.88-7.11) | 6.82 (6.74-7.01) | 0.08 |
| Worst BE within 1 hour of birth, median (range) | -16.0 (-22.5, -12.0) | -17.2 (-20.8, -13.7) | 0.60 |
| Need for ventilation at 10 min, N (%) | 22 (71%) | 11 (65%) | 0.75 |
| Apgar score at 10 min, median (IQR) | 6 (5-8) | 7 (5-8) | 0.59 |
| aEEG abnormalities before TH  Moderately abnormal, N (%)  Severely abnormal, N (%) | 29 (94%)  2 (6%) | 17 (100%)  0 (0%) | 0.53 |

Supplementary Figure legends

**Supplementary Figure 1:** Scatterplot between index of multiple deprivation and general composite score in controls (a) and cases (b).

**Supplementary Figure 2:** Comparison between Structural (X axis) and Pragmatic (Y axis) language scores in controls (a) and cases (b).

**Supplementary Figure 3:** Distribution of Social Interaction Difference Composite between control (a) and case (b) children. Structural language scores were lower than the pragmatic language scores in both controls and case children.
